# Entering a Room

**Published:** 1864-04

**Authors:** 


**Entering a Room.**—I have sometimes envied the coolness and self-possession of those gentlemen who, fortified by long practice, can enter a drawing-room, having no previous knowledge of its inmates, with as much *sangfroid* and indifference as if they were lounging into a box at the opera, and commence a conversation without exhibiting the slightest embarrassment. Yet, after all, I doubt whether they are to be envied, for I apprehend that such demeanor must be the result either of remarkable self-complacency or of callousness of the heart and imagination. It argues the absence, I think, of that chivalrous feeling toward the fair sex which in the middle ages was carried to so extreme a length that, in the words of an old writer of romance, "a true knight should stand more awed and abated in the presence of beauty than if he were summoned before the throne of the most puissant emperor of the world."—*Norman Sinclair.*

